# Tafenoquine lactation pharmacokinetics: a pilot study

**DOI:** 10.1186/s12936-025-05685-z

**Published:** 2025-12-04

**Authors:** Mary Ellen Gilder, Thanaporn Wattanakul, Joel Tarning, Warunee Hanpithakpong, Laaongsri Niwetphongprai, Cindy S. Chu, Gornpan Gornsawun, Wirawatn Tawantochai, Eh Heet, Podjanee Jittamala, Makoto Saito, Nicholas J. White, François Nosten, Germana Bancone, Rose McGready

**Affiliations:** 1 Shoklo Malaria Research Unit (SMRU), Mahidol-Oxford Tropical Medicine Research Unit, Faculty of Tropical Medicine, Mahidol University, 78/1 Moo 5 Mae Ramat, Tak, 63140 Thailand; 2 Centre for Tropical Medicine and Global Health, Nuffield Department of Medicine, University of Oxford, Oxford, UK; 3 Mahidol-Oxford Tropical Medicine Research Unit (MORU), Faculty of Tropical Medicine, Mahidol University, Bangkok, Thailand; 4 Lao-Oxford-Mahosot Hospital Wellcome Trust Research Unit, Vientiane, Lao PDR; 5 Department of Tropical Hygiene, Faculty of Tropical Medicine, Mahidol University, Bangkok, Thailand

**Keywords:** Breastmilk, Malaria, Safety, Vivax, Radical cure

## Abstract

**Background:**

Radical cure with an 8-aminoquinoline is required to prevent relapses from *Plasmodium vivax*. Single-dose treatment with tafenoquine offers significant advantages over primaquine, but the amount of tafenoquine excreted in breastmilk is unknown. As tafenoquine can cause serious haemolysis in G6PD deficient individuals, the amount of drug in breastmilk must be determined in order to advise on safe and effective treatment for lactating women.

**Methods:**

Six healthy lactating volunteers ≥ 1 year postpartum were recruited for this mother-only pilot pharmacokinetic study. Paired breastmilk and venous samples were taken on day 0, 1, 7 and 14. Breastmilk samples were collected in serial 5 ml aliquots to test foremilk and hindmilk drug concentrations.

**Results:**

Tafenoquine concentrations in milk were similar to concentrations in blood (milk/plasma ratio 1.09, range: 0.58–1.63). Concentrations were higher in hindmilk and in lower volume samples. Estimated drug passed to infants was calculated using a standard estimate of milk intake (150 ml per kg per day) and a high intake scenario (200 ml per kg per day). The maximum estimated dose that would be passed to the infant on a single day was 0.050 mg/kg in the first 24 h (range 0.032–0.062 mg/kg). Median weight-adjusted proportion of a therapeutic dose that infants would ingest (relative infant dose, RID) in total ranged from 5.07% (assuming of 150 ml milk per kg per day and 10 mg/kg therapeutic dose) to 13.52% (assuming 200 ml milk per kg per day and 5 mg/kg therapeutic dose). The drug was well tolerated by mothers.

**Discussion:**

This pilot study demonstrated measurable tafenoquine in breastmilk, with very small amounts of drug passing to babies on any given day but overall estimated RIDs around the conventional 10% threshold for safety concerns. Further studies are needed to determine with greater certainty the excretion of tafenoquine in breastmilk and drug concentrations in infant blood to inform recommendations for breastfeeding women.

**Supplementary Information:**

The online version contains supplementary material available at 10.1186/s12936-025-05685-z.

## Background

Radical cure with 8-aminoquinoline drugs, currently either primaquine or tafenoquine, is needed to prevent relapse of *Plasmodium vivax*, the most common cause of malaria outside of Africa. The primary advantage of tafenoquine is its slow elimination, providing radical cure with a single dose which circumvents the challenge of adherence to 7–14 day courses of primaquine. However, use of both drugs is hampered by the potentially severe haemolysis they trigger in individuals with glucose-6-phosphate dehydrogenase (G6PD) deficiency, which is an X-linked disorder and the most common enzyme deficiency in the world [1]. Tafenoquine may cause severe haemolysis in individuals with < 70% of normal G6PD activity [2], and treatment is withheld in males and females with severe G6PD deficiency and heterozygous females if they have G6PD activity below this threshold. This excludes > 25% of the population from tafenoquine radical cure in areas with a high prevalence of G6PD deficiency [3].

As the G6PD status of a fetus cannot be tested, the 8-aminoquinoline drugs are contraindicated in pregnancy and pregnant women cannot benefit from radical cure. Safety of radical cure given after delivery is contingent on the amount of drug passed to nursing infant. Characterizing drug exposure to a breastfed infant is, therefore, essential. Minimal concentrations of primaquine are found in mature breastmilk, providing evidence of safety for breastfed infants at least one month old [4] and the World Health Organization (WHO) updated their guidance in 2024 to reflect this evidence [5].

Tafenoquine pharmacokinetics have been well characterised in adults [6, 7] and children [8]. Peak concentrations are reached approximately 12–15 h after dose and the plasma elimination half-life is about 15 days. It is extensively distributed, has very high levels of protein binding (99.5%) [9] and lipid solubility (estimated logP 5.57) [10], and undergoes slow metabolism. The full extraction profile is unknown, but tafenoquine represents the major drug-related compound in human plasma and renal elimination of unchanged tafenoquine is low.

There are no published data on tafenoquine excretion in breastmilk. Physiologically-based pharmacokinetic (PBPK) models can be helpful to fill data gaps with informed estimates, but their predictive accuracy for drug excretion in breastmilk is mixed. In a recent publication, two PBPK models used to estimate the excretion of tafenoquine in breastmilk produced estimates that differed by an order of magnitude [10]. Direct pharmacokinetic data is needed to provide confident estimates of infant exposures through breastmilk.

Breastmilk composition is dynamic, changing over the course of a single feed (foremilk and hindmilk) [11], and changing over the long term (from colostrum, transitional milk, mature milk and finally to weaning milk) [12]. For a lipophilic drug like tafenoquine, two important factors are likely to affect breastmilk concentrations: the degree of “leakiness” of the junctions between the alveolar cells in the breast [13], and the fat content in the breastmilk [11]. Generally, the junctions between alveolar cells are most open when breastmilk volumes are lowest (colostrum, weaning milk and during episodes of mastitis). Fat content is lower in the first milk of a feed (foremilk), and increases at the end of the feed (hindmilk) as the breast nears emptiness. Fat content also peaks when breastmilk volume is lower (in the evening, in cases of low milk production, and during weaning). Thus, although drug transfer may be higher at these times, the volume of milk ingested is lower so clinically relevant increases in infant exposure compared with high breastmilk production states is still unlikely. The greatest number of adverse events attributed to drug exposure in breastmilk happens when infants ingest the largest volume of milk relative to their body weight [14], which generally occurs between days 4 and 35 of life [15].

The primary objective of this study was to obtain preliminary data to estimate potential tafenoquine exposure for the breastfed infant to inform risk assessment and the design of future detailed pharmacokinetic studies, and provide seed data for modelling and simulation. Secondary objectives included determining concentrations of tafenoquine in foremilk (first milk in a feed) and hindmilk (last milk in a feed), and optimization of breastmilk sampling procedures and haematological monitoring.

## Methods

### Setting

This study was conducted at the Shoklo Malaria Research Unit (SMRU) located on the Northwestern border of Thailand, an area of low seasonal malaria transmission. SMRU clinics provide free inpatient and outpatient services to a population of migrant workers and minority communities resident along the Thailand-Myanmar border with a focus on maternal and child health, including antenatal, delivery and neonatal care. During the time of the study, SMRU’s work contributed to the near elimination of *Plasmodium falciparum* malaria so *P. vivax* is now the most prevalent malaria species in the local communities.

### Study recruitment and consent

Women presenting with young children for outpatient, vaccine or contraception services were asked about breastfeeding plans. Mothers of children at least one year old who planned to stop breastfeeding were counselled about the study, and written informed consent was obtained.

### Study design

This was a prospective open-label, single site, mother-only lactation pharmacokinetic pilot study with a sample size of six mothers. Volunteers received tafenoquine (60 Degrees Pharmaceutical, 300 mg single dose, i.e. 6 mg/kg for a 50 kg woman) after a meal of their choice.

Women were eligible for the study if they were at least 16 years old, were nonpregnant and willing to delay pregnancy for the subsequent 90 days. Women were excluded if they were known to be hypersensitive to 8-aminoquinolines, had G6PD activity < 70% of the normal male population median as assessed by spectrophotometry [16], had serious conditions that would place the participant at undue risk or interfere with the results of the study, had an ALT > 2 × the upper limit of normal, were anaemic (haematocrit < 33%), had known history of severe jaundice in a previous child, had a history of psychiatric illness or abnormal depression screening score, or had received a blood transfusion within the 3 months before screening. Mental health screening was done using a locally validated screening tool (adapted from DSM IV-SCID) [17, 18] along with a comprehensive medical history.

Because of practical challenges ensuring that a breastfed toddler did not continue to breastfeed after planned breastfeeding cessation, infants’ G6PD activity status was reviewed using records of the routine G6PD screening test done at birth (STANDARD G6PD Analyzer, SD Biosensor). For infants not screened at birth, a capillary sample was used to measure G6PD activity by biosensor. All had activity ≥ 70% of normal.

### Baseline and follow up laboratory methods

Screening after consent included malaria smear, urine pregnancy test, G6PD testing by biosensor and spectrophotometry, kidney function tests and liver enzymes, and a full blood count. Safety monitoring for adverse events related to the study medication was done at every sampling timepoint and on one additional visit on day 4. This included field haematocrit and haemoglobin (HemoCue) measurements, full blood count, peripheral blood smear markers for haemolysis (e.g. reticulocytes, schistocytes, bite cells), and non-invasive methaemoglobin measurement using a Masimo Radical 57 oximeter.

G6PD spectrophotometric reference assay was performed using Pointe Scientific kits (assay kit # G7583-180, lysis buffer# G7583-LysSB). Kinetic determination of G6PD activity at 340 nm was performed using a SHIMADZU UV-1800 spectrophotometer with temperature-controlled cuvette compartment (30°C) [16]. Samples were analysed in duplicate and mean activity was expressed in IU/gHb using the haemoglobin concentration obtained by full blood count analysis. The final result was calculated using manufacturer’s temperature control factor of 1.37. Two controls (normal, intermediate or deficient; Analytic Control Systems, USA) were analysed at every run and results compared with expected ranges provided by manufacturer. Reference G6PD normal values were obtained before [16]. Details about other laboratory methods are included in the supplementary file 1.

### Pharmacokinetic sampling and analysis

Women enrolled in the pilot study had paired venous blood and breastmilk samples drawn during 5 sampling windows after taking tafenoquine: 8–15 h, 16–24 h, 30–42 h, 6–8 days, and 12–16 days. Samples for measurement of venous drug concentrations were taken in 2 ml EDTA tubes from which plasma was removed after centrifugation at 1500–2000 g for 10 min. To elucidate differential drug excretion in foremilk and hindmilk, women were asked to express breastmilk in 5 ml numbered aliquots from the fullest breast at all timepoints. If the total volume was ≥ 25 ml, samples were taken from the first and last containers. Remaining milk was combined and mixed thoroughly before taking aliquots of mixed milk. If the total volume was < 25 ml, all the available milk was mixed thoroughly and only mixed milk samples were taken as the difference between foremilk and hindmilk at smaller breastmilk volumes is expected to be less [19].

Samples were frozen in liquid nitrogen within 1 h and transported in liquid nitrogen to SMRU’s central lab where they were stored at below −70 °C before transfer on dry ice to Bangkok for pharmacokinetic measurements. Pharmacokinetic drug measurements of tafenoquine were performed at the Department of Clinical Pharmacology, Mahidol-Oxford Tropical Medicine Research Unit, Bangkok, Thailand. Tafenoquine concentrations were quantified by liquid chromatography coupled with tandem mass spectrometry (LC–MS/MS) using validated methods in plasma and breastmilk.

The LC–MS/MS method for the determination of tafenoquine was developed and validated over concentration ranges of 6.08–1250 ng/mL using 100 mL of EDTA plasma and 100 mL of human breastmilk. The bioanalysis method was validated in each specific biological matrix. The LC MS settings were identical for both plasma and breast milk while the sample clean up procedures were developed for each matrix. Both methods used phospholipid removal plate, Phree Phenomenex, with different precipitation solutions: plasma was precipitated by 1% acetic acid, breast milk was precipitated by 0.1% formic acid. Separation was then achieved using a reverse phase column (4.6 mm by 50 mm, 3.5 mm; Agilent, USA) with a binary gradient solvent system consisting of 20 mM ammonium formate and acetonitrile. Mass detection was performed using a triple-quadrupole mass spectrometer (API5000; AB Sciex, Foster City, CA, USA) operating in positive electrospray ionization mode. The transition m/z (mass to charge ratio) of tafenoquine (464.0 to 379.3) was monitored using multiple-reaction monitoring (MRM). The method did not show any signs of severe ion suppression or enhancement.

The within-day and between-day accuracy and precision at all quality control levels from EDTA plasma were below 10%. The coefficients of variation were lower than 7% in all QC samples during analysis clinical samples. The within-day and between-day accuracy and precision at all quality control levels from human breastmilk were below 15%. The coefficients of variation were lower than 2% in all QC samples during analysis clinical samples.

### Statistical and pharmacokinetic analysis

A noncompartmental analysis of drug concentration–time data was performed using PKanalix 2024R1. The milk/plasma ratio (M/P) was calculated with paired samples at each time point for each participant, and as a ratio of the area under the time-concentration curve (AUC) for milk to the AUC for plasma. The AUC ratio is preferred for medications with short elimination half-lives, but the best measure for medications that are eliminated over several weeks is not addressed in the literature. The plasma and breastmilk AUC between the first and last measurement was used for M/P ratio because of uncertainty extrapolating to infinity given the limited sampling schedule.

The word “Infant” is used here to refer to any breastfeeding child to facilitate comparisons between the categories of data presented and other lactation pharmacokinetic studies. The infant daily dose estimates the amount of drug ingested by the breastfed child in breastmilk in a 24 h period. Relative infant dose (RID) is a measure of the estimated weight-adjusted dose of the study medication that the child is exposed to via the breastmilk in comparison to a therapeutic dose [13]. Relative infant dose of 10% has been accepted as a threshold for concern, with > 25% likely unsafe. While convenient, these thresholds are arbitrary and not based on any empirical evidence [13].

For details of equations used to calculate daily and relative infant doses, see the supplementary file 2.

## Results

Six women were enrolled in the pilot study in June 2022, of whom five women completed all sampling timepoints (Table 1). Median actual weight was 51 kg and tafenoquine 300 mg was generally well tolerated. One woman had a previous *P. vivax* infection without subsequent radical cure, while the others were healthy volunteers. There were no clinically significant decreases in Hb (median (range) decrease −0.6 g/dl Hb (0 to − 1.7)) or HCT (− 4% HCT (0 to − 6)). HCT and Hb minima were seen on median day 1 (range 0–14). Reticulocytes were stable with a median (range) increase of 0.6% (0–1.3) with a maximum on a median day 5 (0–14).

**Table 1:** Baseline and safety characteristics – all presented as median (range)

Characteristic	median (range)
Age, year	36 (23–42)
Weight, kilogram	51 (37–62)
Height, centimetre	151.55 (149–155)
Number of children	3 (1–9)
Child age, months	17 (12–28)
Baseline Haematocrit†, %	39 (38–43)
Baseline haemoglobin†, g/dL	12.1 (11.3–13.4)
Baseline creatinine, mg/dL	0.6 (0.6–0.8)
Baseline alanine transaminase, U/L	14.4 (8.5–30.7)
Haematocrit nadir†, %	35 (33–39)
Haemoglobin nadir†, g/dL	11.7 (10.3–12.0)
Haematocrit day 14†	39 (37–42)
Haemoglobin day 14†	12.7 (12.0–13.6)
Methaemoglobin peak†, %	3 (1.7–4.3)
Methaemoglobin peak day†	7 (7–14)

^†^ includes 5 women with complete follow up to day 14

*g/dL* gram per decilitre, *U/L* units per litre

Haematological parameters on day 14 were similar to baseline values. G6PD activity detected in single red blood cells by fluorescence-activated cell sorting (FACS) [20] was stable throughout treatment for individual women. Detailed results of Hb, reticulocytes and Heinz bodies are included in the supplementary file 3.

Adverse events were mild including dizziness and slight increases in methaemoglobin (Table 1). There were no severe adverse events. Mental health screening before and after the medication was unchanged. The sixth woman withdrew from the study for reasons unrelated to the study and she was well at the end of the study period (day 14). No participants became pregnant within 90 days of TQ dose.

### Pharmacokinetic results

A summary of the estimated pharmacokinetic parameters from the noncompartmental analysis is provided in Table 2. Overall, peak concentrations for tafenoquine in both breastmilk and plasma were observed at 16 (milk range 12–36) hours, with a maximum concentration in breastmilk of 308 (range 266–351) ng/ml. Concentrations in milk and plasma were similar, with an overall median milk/plasma (M/P) ratio by AUC (AUC_0-last,milk_/AUC_0-last,ven_) of 1.09 (0.58–1.63) and median M/P ratio form paired samples of 0.89 (0.17–1.57).

**Table 2:** Noncompartmental analysis pharmacokinetic parameters for venous plasma and breastmilk tafenoquine concentrations

	Plasma		Mixed Breastmilk	
	Median	Range (min–max)	Median	Range (min–max)
T_max_ (h)	16	12–20	16	12–36
C_max_ (ng/mL)	290	224–329	308	266–351
AUC_d0-d14_ (h × ng/mL)*	57,502	52,032–62,605	58,322	13,453–80,112
AUC_d0-d30_ (h × ng/mL)*	77,748	66,009–86,294	70,331	46,101–72,729
AUC_0-inf_ (h × ng/mL)*	88,040	70,677–101,595	72,353	51,773–77,949
M/P ratio by AUC (AUC_0-last,milk_/AUC_0-last,plasma_)*			1.09	0.58–1.63
M/P ratio (from paired samples)			0.89	0.17–1.57
M/P ratio at hour 12			0.99	0.66–1.51
M/P ratio at hour 20			0.87	0.45–1.57
M/P ratio at hour 36			0.89	0.17–1.45
M/P ratio at day 7*			0.99	0.50–1.55
M/P ratio at day 14*			0.69	0.42–0.90

^*^Excludes one participant who withdrew after hr 36

*T*_*max*_ Time of maximum concentration, *C*_*max*_ maximum concentration, *AUC* area under the time-concentration curve, *d* day, *inf* infinity, *h* hour, *ng/mL* nanogram per mililitre, *M/P* milk to plasma concentration ratio

**Fig. 1:**
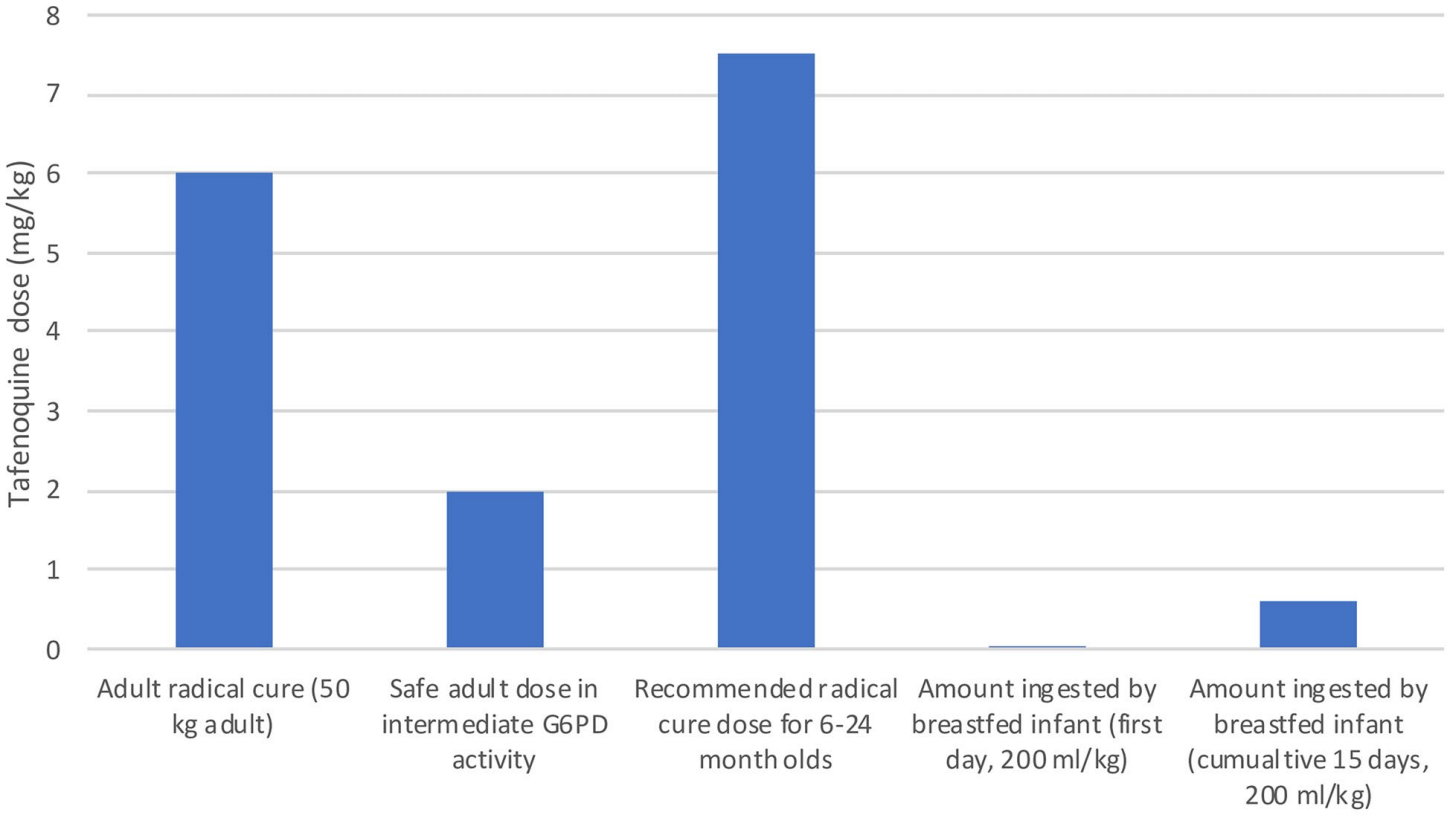
Estimated dose of Tafenoquine that a breastfed infant would be expected to ingest through breastmilk compared with maternal dose for a hypothetical 50 kg woman (mg/kg) calculated using non-compartmental analysis. The maximum milk intake (200 ml/kg) is used as to show the scenario with the maximum drug ingested by a breastfed infant. The 100 mg (2 mg/kg) dose which did not cause haemolysis in women with intermediate G6PD activity (40–60% of normal) [21], and 7.5 mg/kg which falls in the middle of the range of proposed infant and paediatric doses [8] are included for comparison

The highest dose that the infant would ingest in breastmilk in a single day (absolute infant dose for day 0 at the maximum infant intake of 200 ml breastmilk/kg/day) was estimated to be 0.050 (range 0.032–0.062) mg/kg/day, which corresponds with an RID of 0.83% (0.53–1.04) of the weight-adjusted maternal therapeutic dose (Fig. 1, Table 3). Maximum cumulative exposure over 14 days was estimated to be 0.486 mg/kg, or 8.10% (4.66–8.56) of the maternal dose (using 200 ml/kg/day). The majority of exposure is expected to be in the first week.

**Table 3:** Estimated dose to the breastfed child

	150 ml/kg/day		200 ml/kg/day	
	Median	range	Median	range
Estimates of absolute infant dose				
Infant daily dose_d0_ (mg/kg/day)*	0.037	0.024–0.047	0.050	0.032–0.062
Infant daily dose_d1_ (mg/kg/day)*	0.033	0.006–0.047	0.044	0.008–0.062
Infant daily dose_d7_ (mg/kg/day)*	0.024	0.013–0.038	0.031	0.018–0.051
Infant daily dose_d14_ (mg/kg/day)*	0.010	0.007–0.011	0.014	0.010–0.015
Total infant dose/Body Weight/14 days (mg/kg) ~	0.365	0.210–0.385	0.486	0.280–0.514
Average infant daily dose/Body Weight/day_d0-15_ (mg/kg/day) ~ ~	0.024	0.014–0.026	0.032	0.019–0.034
Total infant dose/Body Weight/30 days (mg/kg) ~	0.473	0.311–0.539	0.631	0.415–0.719
Total infant dose/Body Weight/75 days (mg/kg) ~	0.507	0.371–0.630	0.676	0.494–0.840
Average daily infant dose/Body Weight/day _d0-75_ (mg/kg/day) ~ ~	0.007	0.005–0.008	0.009	0.007–0.011
Estimates of relative infant dose (RID)				
RID per day (average) (%)†	0.113	0.082–0.140	0.150	0.110–0.187
RID_maximum day_ (%)†	0.624	0.394–0.780	0.832	0.525–1.040
RID_d0-14_ (%) †	6.075	3.497–6.422	8.100	4.662–8.563
RID_total exposure_ (%)†	8.449	6.178–10.499	11.265	8.238–13.999

*mg/kg* milligram per kilogram, *d* day, *RID* relative infant dose

Daily volume of 150 ml/kg/day is the standard estimated breastmilk intake for an infant, while 200 ml/kg/day represents a maximum exposure scenario

See supplemental file for details of calculations: * Eq. 1; ~ Eq. 2; ~  ~ Eq. 2/number of days; † Eq. 3 (using maternal therapeutic dose of 6 mg/kg as denominator)

Estimates of the RID were affected by both the estimated milk volume and the dose used as a proxy for the therapeutic dose for the breastfed child (therapeutic doses for children < 2 years are not yet established). Therapeutic doses tested in Phase 2b trials range from 5 to 10 mg/kg for children (mean 7.5 mg/kg) [8]. RIDs were highest when using 5 mg/kg, and decreased as the presumed therapeutic dose increased to 10 mg/kg giving a range of estimated RID from 5.07% to 13.52% (supplementary file 4).

### Drug concentrations in breastmilk: effect of milk volume

**Fig. 2:**
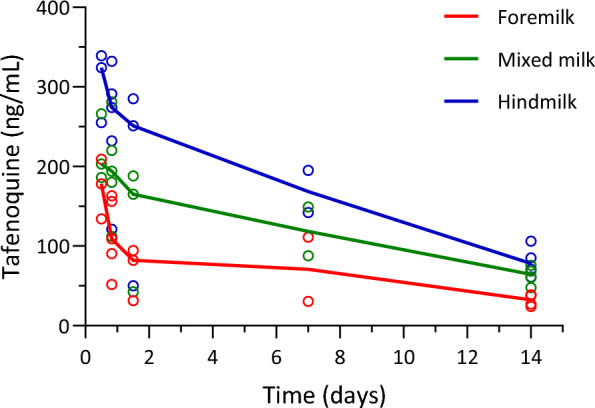
Foremilk, hindmilk and mixed milk tafenoquine concentrations in samples ≥ 25 ml. Vertically aligned dots represent aliquots from the same time point. Foremilk consistently had lower tafenoquine concentrations than hindmilk, while whole breast mixed milk concentrations fell between hindmilk and foremilk, as expected

Five of the six women had at least one time point with at least 25 ml of milk expressed, for a total of 17 time points with foremilk and hindmilk comparisons (supplementary file 4). Median milk volume for these time points was 50 ml (range 24–104). Concentrations were consistently higher in hindmilk (with high fat concentrations) and lower in foremilk, with a median concentration ratio of 2.42 (IQR 1.62–3.03) for paired samples (Fig. 2).

Differences between tafenoquine concentrations in foremilk and hindmilk increased with increasing volumes of total expressed breastmilk, with a 38% (95% CI 13–63%) increase in the difference between the foremilk and hindmilk concentrations for every 10 ml increase in volume (p = 0.005). Hindmilk concentrations were a median of 3.85 (IQR 3.02–5.53) times higher than foremilk for the four samples that were over 75 ml. Likewise, drug concentrations in aliquots of mixed milk taken from a larger volume of total milk were lower relative to time-matched plasma samples from the same participant (lower M/P ratios) than aliquots taken when breastmilk production was lower and only small volumes of milk could be expressed (supplementary file 5, Fig. 3).

**Fig. 3:**
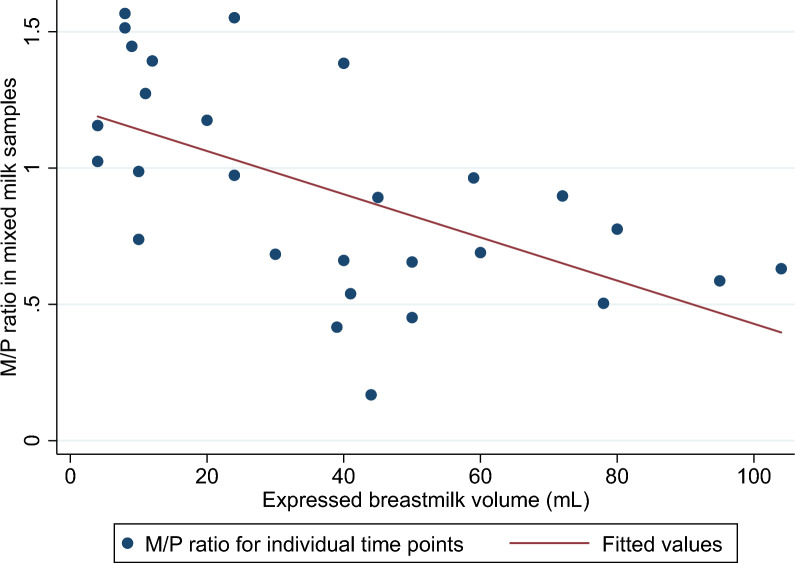
The relationship between the ratio of the breastmilk tafenoquine concentrations to mother plasma tafenoquine concentrations (M/P ratio) and expressed breastmilk volume. As milk volumes increase, milk tafenoquine concentrations fall in comparison with plasma tafenoquine concentrations. Each dot represents the M/P ratio of a pair of milk and plasma samples from an individual participant at a certain time point

## Discussion

This pilot study provides preliminary data on the pharmacokinetics of tafenoquine in lactation. As with most drugs taken while breastfeeding, concentrations in breastmilk were measurable, and estimated amount of drug ingested by the infant was an order of magnitude lower than that of adults. However, data on more women in different phases of lactation and drug concentrations in breastfeed infants are needed to draw conclusions about safety of this long-acting drug during breastfeeding.

Based on the preliminary data here, we estimate that the total amount of tafenoquine that an infant would ingest over 75 days would be around 0.5–0.7 mg/kg. Determining the clinical significance of this amount is complicated. Haemolysis in G6PD deficient individuals due to 8-aminoquinolines has been extensively studied and is known to be dose dependent in adults [22]. A drug challenge study giving varying doses of tafenoquine to adult volunteers with intermediate G6PD activity (40–60% of normal; G6PD Mahidol) found that 100 mg (e.g. 2 mg/kg for a 50 kg adult) of tafenoquine did not cause significant haemolysis [21]. Estimated mg/kg doses recommended for infants and young children are higher than for adults (as for many other drugs), but trials did not actually include children under 2 years old [8]. Younger children (including those with class B G6PD deficiency variants) experienced larger drops in haemoglobin than adults when exposed to 0.25 mg/kg primaquine, though no infants or young children in that study developed severe anaemia or required blood transfusion [23, 24]. A retrospective study of primaquine administration to infants < 6 months was mostly reassuring but had some mixed safety signals [25]. Importantly, increased haemolysis in the first two weeks of life could contribute to neonatal jaundice and potentially kernicterus. A complete pharmacokinetic study including infant plasma pharmacokinetic measurements and measurement of drug concentrations in different phases of lactation is needed to establish the extent of infant exposure and estimate the potential risk to breastfeeding babies who are fully or partially G6PD deficient.

Drug concentrations in milk in this study were generally close to that of plasma, an unexpected finding for a highly protein bound drug [13]. However, these findings align with the lower range of milk concentrations predicted by PBPK modelling, highlighting the complex dynamics of drug transfer into milk [10]. In this case, the influence of lipophilicity appears to be important, as seen in the comparison of foremilk and hindmilk concentrations.

The differences in drug concentrations in foremilk and hindmilk, as well as large and small breastmilk volumes have important implications for lactation pharmacokinetic studies. The US FDA’s draft guidance on lactation pharmacokinetic studies recommends collection of all milk from both breasts for a 24 h period [26]. However this guidance is rarely followed, and has been considered burdensome [27]. Complete expression of one breast at each time point has the benefit of sampling a close to physiological distribution of foremilk and hindmilk without undue burden to the participants, or disruption of breastfeeding in studies where direct feeding is continued. Mixing of pre- and post-feed samples has been suggested as an alternative to whole breast sampling [27, 28]. This study suggests that such a method might be appropriate for tafenoquine as the mixed milk concentrations fell evenly between foremilk and hindmilk concentrations, however, this relationship may differ for different drugs. This method is also dependent on the infant taking a typical full feed at the moment when the samples are to be collected (best achieved with flexible sampling windows with exact time points cued by infant hunger). If investigators choose to use pre- and post-feed aliquots, it is advisable to test foremilk, hindmilk and concentrations in milk from a fully emptied breast in a few samples collected near expected peak concentrations to confirm the appropriateness of this approach before embarking on a complete lactation PK study. Measurement of breastmilk fat (creamatocrit) would help clarify the relationship between fat and drug concentrations, and should be considered in pharmacokinetic studies of lipid soluble drugs.

Importantly, use of a convenient 5–10 ml random breastmilk sample, which is operationally easy, would have dramatically underestimated the total exposure through the milk. Women, if asked to provide a breastmilk sample, will naturally express their fuller breast (predominantly foremilk). If such a sampling approach was used in this study, it might have underestimated the drug passed to the infant by 50%.

If a breastfeeding woman taking tafenoquine wanted to decrease infant exposure, switch feeding (short feeds on alternating breasts) or feeding only milk pumped early in a pumping session would be options to minimise the amount of hind milk ingested by the baby. For mothers who pump, pumped hind milk could be diluted with drug-free milk (e.g. pumped prior to taking tafenoquine) or discarded. Any of these techniques, while preferable to interruption of breastfeeding, could have negative impacts on milk production, and infant growth, nutrition and satiety. If these techniques are recommended, they should be paired with clinical support to minimise adverse effects on the mother (mastitis, pump trauma, hyper- or hypolactation) or infant (poor weight gain) especially in low-resource settings where maintenance of healthy lactation is essential.

As breastfeeding frequency declines in later lactation, decreasing volumes of milk contain higher proportions of fat and protein, and tight junctions preventing transfer of drugs into breastmilk open [12]. This suggests that lactation pharmacokinetic studies recruiting participants at the end of lactation, including the current study, likely overestimate the drug exposure to breastfed infants by extrapolating the drug concentrations in this high-fat weaning milk to the high volumes of milk produced in the first months (after the first 1–2 weeks). In this study, the overall milk/plasma ratio in samples with volumes < 25 ml was more than double that of samples > 75 ml. Feed volumes during the critical early months, when the majority of adverse events attributed to lactational transfer of drugs occurs, would be expected to be 60–90 ml [14], which could result in substantially lower drug concentration in milk. Overall, these considerations underscore the value of conducting lactation pharmacokinetic studies under situations that mimic as closely as possible the breastfeeding scenarios in which the drug will be used. In the case of this study, concerns about infant safety resulted in the limited sampling schedule and mother-only, late-lactation design.

Finally, there is little precedent for interpretation of RID and other lactation pharmacokinetic safety parameters for drugs with very long half-lives. Studies have variably reported total amount of drug ingested by the breastfed baby [29] or average daily amount ingested by the baby [30]. Continuous dosing of short-lived 8-aminoquinolines transiently decreases susceptibility to haemolysis in individuals with class B G6PD mutations because haemolysis of older red blood cells triggers a reticulocyte response resulting in a population of younger red blood cells with higher G6PD activity [31]. Thus, it is likely that the total infant dose ingested in breastmilk (including increasingly trivial doses beyond the first week) is less important than the maximal dose received by the infant in a single day.

There are limitations to this pilot study: a small number of volunteers were studied and not all completed the study, there were no child drug concentrations available, and only sparse sampling of late lactation milk was used. Potentially significant changes to breast milk composition, enzyme maturity, and feeding patterns between infancy and later lactation could affect infant exposure. Noncompartmental analysis was used in this preliminary report of pilot data, but this approach has limitations, particularly with sparse pharmacokinetic sampling and tafenoquine’s long half-life. Model-based estimation of pharmacokinetic parameters based on the data presented here would add valuable information about the robustness of these estimates, as would collection of more clinical data. An additional limitation was the absence of data on breastmilk biomarkers such as sodium (a marker for the openness of junctions between alveolar cells) [32], or creamatocrit to confirm the hypothesized factors influencing breastmilk drug concentrations. Many important aspects of human lactation remain incompletely described in the literature, and much can be gained through greater attention and support for this research.

## Conclusions

This pilot study produced preliminary measurements of tafenoquine in breastmilk. Estimated amount of tafenoquine that would pass to the infant in breastmilk is small but not entirely negligible, supporting the importance and the safety of including actively breastfeeding mother-infant dyads in future detailed pharmacokinetic studies. Lactation pharmacokinetic study sample collection should represent physiologically distinct phases of breastmilk, including foremilk and hindmilk, in order to produce reliable results and should include creamatocrit. Haematological monitoring with haemoglobin, haematocrit, reticulocytes and methaemoglobin appeared to be the most meaningful measures and will likely be meaningful tools to monitor infants exposed to tafenoquine in future breastfeeding studies.

## Supplementary Information

Supplementary material 1

## Data Availability

Data cannot be shared publicly because of the sensitivity of data for this population of undocumented refugees and migrants. De-identified participant data are available from the Mahidol Oxford Tropical Medicine Data Access Committee upon request from this link: https://www.tropmedres.ac/units/moru-bangkok/bioethicsengagement/datasharing.
