# Supplementary material for: Tafenoquine lactation pharmacokinetics: a pilot study
Source: Malar J. 2025 Dec 4;25:15. doi: 10.1186/s12936-025-05685-z (PMC12781337; doi:10.1186/s12936-025-05685-z)
Supplement: Supplementary file 1 — Supplementary material 1 [file 12936_2025_5685_MOESM1_ESM.docx]

**Tafenoquine lactation pharmacokinetics: a pilot study**

**Supplementary files:**

**Supplementary file 1 – Estimation of lactation pharmacokinetic parameters**

Calculations: Daily infant dose (quantity of the drug ingested in 24 hours) on individual days was estimated using *Equation 1.* Daily milk volume was estimated at 150 ml/kg/day (by convention), and a hypothetical maximum of 200 ml/kg/day.[1,2]

$Daily infant dose (ng)=Average concentration in breast milk \left( \frac{ng}{mL} \right)\times daily milk volume(mL)$ Eq.1

Since most days had only one sampling time point, and the elimination of tafenoquine is slow, the concentration at that time point was used as the average breastmilk concentration for that day. On day 0, the mean of the two measured concentrations (from the time windows of 8-15 hours and 16-24 hours) was used.

Because of the slow elimination half-life of tafenoquine (estimated at 15 days),[3] infant exposure in milk was also calculated based over longer periods of N days (N = 14, 30, and 75; 1, 2 and 5 half-lives) using *Equation 2 (simplified):*

$Total infant dose over N days (ng/kg)=\frac{{AUC}_{milk} \left( h\times\frac{ng}{mL} \right)}{{AUC}_{venous} \left( h\times\frac{ng}{mL} \right)}\times\frac{{AUC}_{venous N} \left( h\times\frac{ng}{mL} \right)}{24\frac{h}{day} \times N days}\times milk vol (\frac{mL}{kg*day})\times N days$

$Total infant dose over N days (ng/kg)=\frac{M}{P}ratio\times\frac{{AUC}_{venous N} \left( h\times\frac{ng}{mL} \right)}{24\frac{h}{day}}\times milk vol (\frac{mL}{kg*day})$ Eq. 2

This approach was chosen instead of calculating more directly from AUC_milk_ because of the greater variability in the milk concentrations (presumably largely due to variability in milk fat composition) compared to venous concentrations. Resulting estimates did not differ dramatically.

The relative infant dose was calculated using *Equation 3*.

$Relative infant dose (\%)=\frac{Infant dose (\frac{mg}{kg})}{Pediatric therapeutic dose (\frac{mg}{kg})}\times100$ Eq. 3

For the primary analysis, the maternal dose of 6 mg/kg (300 mg for median 50 kg women) was used in lieu of the paediatric dose, as there is no established dose for this age group. However, to estimate the range of possible RIDs, a paediatric therapeutic dose of 7·5 mg/kg was used, the mean mg/kg dose of the published proposed paediatric dosing weight bands.[4] In addition, RID was calculated over the range of studied doses from 5-10 mg/kg.

**Supplementary file 2 – Detailed laboratory methods**

Malaria Smear: Parasite count (thin and thick films stained by Giemsa’s method) were read by trained laboratory technicians who undergo regular quality control. Smears were declared negative after 200 high power fields were read.

A urine β-HCG pregnancy test was performed using Bioline HCG test strips or equivalent.

The STANDARD G6PD Analyzer (SD Biosensor, Republic of Korea) was run according to manufacturer’s instruction by collecting 10uL of blood using a EZI-tube and placing it on the device strip. The G6PD activity (U/gHb) and haemoglobin concentration (g/dL) were read from the screen within 2 minutes.

Biochemical analysis was done on serum and haemoglobin typing on EDTA-treated whole blood, both at an accredited external laboratory.

Field blood hematocrit was measured on anticoagulated blood capillary tubes, centrifuged at 10,000 RPMs for 3 minutes and assessed by trained clinic laboratory technicians using a Hawksley Micro-Haematocrit reader. Field haemoglobin was measured using a HemoCue Hb 301 system.

Full blood count (White blood cells and differential count, Red blood cells and platelets indices) were assessed by automated haematology analyzer CeltacF MEK-8222K (Nihon Kohden, Tokyo, Japan) on EDTA-anticoagulated venous blood. Three-levels quality controls were run every day and device maintenance and calibration were performed regularly. Blood smear for red blood cell morphology was stained with Wright’s stain and read according to SMRU SOPs. Reticulocyte counts were determined either by standard slide microscopy on 1000 red blood cells stained with new Methylene Blue or by flow cytometry using Thiazole Orange staining. Heinz bodies counts were determined after staining of RBCs with 10% Crystal Violet.

Whole blood or packed RBC specimens were characterized for intracellular G6PD activity by flow cytometry (FACS) as described previously Shah et al. 2012. Specimens were analyzed using an Accuri™ C6-UV flow-cytometer (BD Biosciences) with 10,000 events recorded in the FL1 channel 533 +/− 30 nm. The relative proportion of G6PD normal and G6PD deficient RBCs were calculated by analysis of distribution of events with different fluorescence using Flow-Jo software.

**Supplementary file 3 – Detailed Haematological results**

Analysis of haemoglobin:

While there were fluctuations in haemoglobin during the study period, there was no evidence of clinically significant haemolysis in any of the participants. Participant #001 (light blue) had a decline in both haemoglobin and haematocrit at day 1 with subsequent rapid return to baseline. Participant #002 (maroon) had beta-thalassemia trait which might be associated with increased intraerythrocytic oxidative stress.

Fig.S1 Time-course of haemoglobin in women receiving tafenoquine


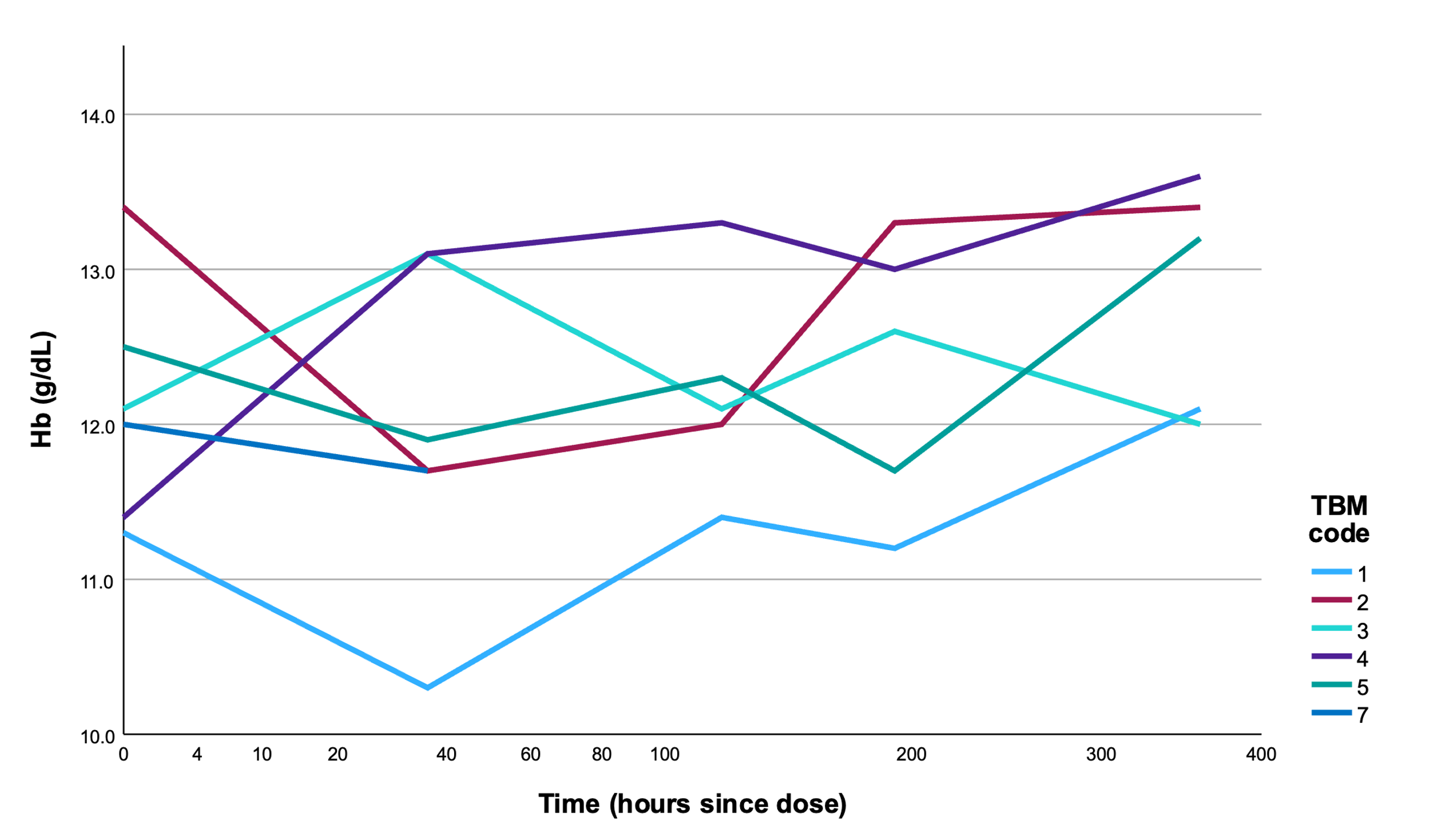


Time is represented in power scale to visualize more clearly repeated samplings in the first 36 hours

Analysis of reticulocytes:

Reticulocyte count did not change during the treatment course except in participant #002 (maroon) where it peaked at 3.9% at day 5 of treatment.

Fig.S2 Time-course of reticulocyte count in women receiving tafenoquine


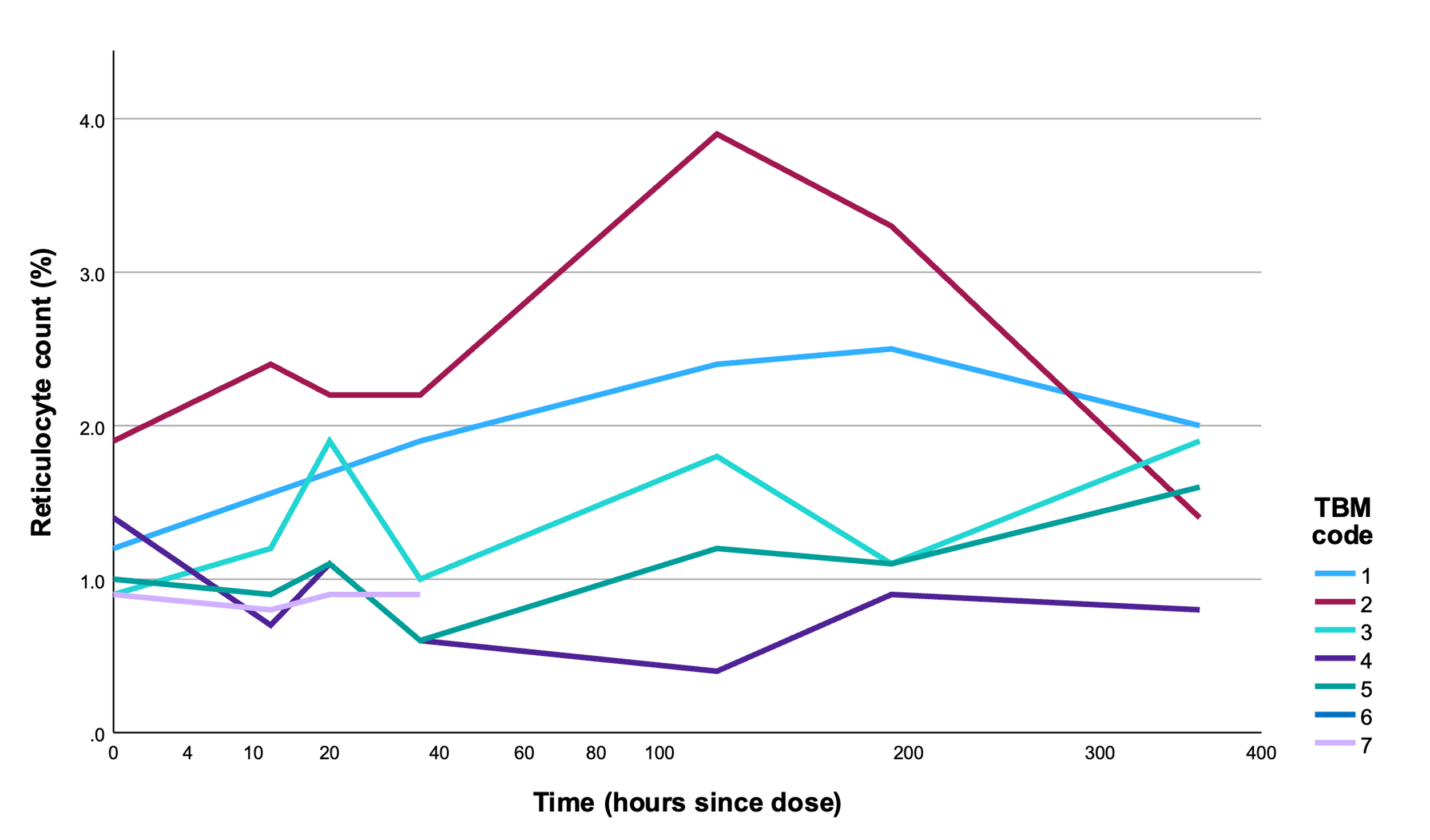


Time is represented in power scale to visualize more clearly repeated samplings in the first 36 hours.

Analysis of Heinz Bodies inclusions into red blood cells:

Percentage of red blood cells presenting with Heinz Bodies inclusions did not change during the treatment course except in participant #002 (maroon) where it reached almost 2% at 10h after treatment.

Fig.S3 Time-course of Heinz Bodies-containing red cells in women receiving tafenoquine


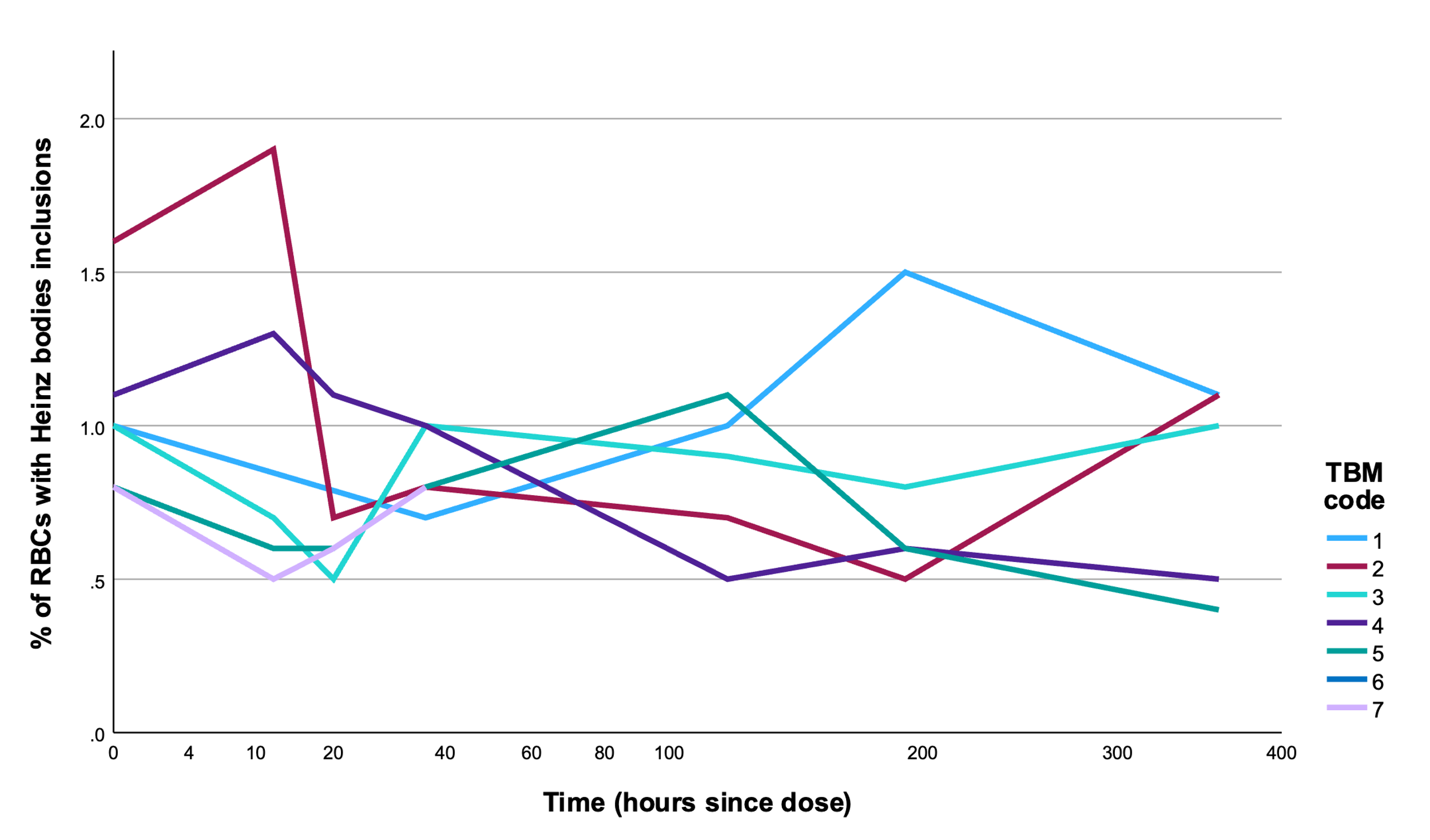


Time is represented in power scale to visualize more clearly repeated samplings in the first 36 hours.

**Supplementary file 4 – Estimated doses ingested by breastfed infant over different time periods and scenarios**

**Table S1 Range of estimated relative infant doses (RID)** The following table shows the range of estimates when using different doses as denominator and different estimated milk volumes. Doses range from 5-10 mg/kg, including 6 mg/kg actual average maternal dose in this study, and 7·5 mg/kg which is the average suggested paediatric dose for this age range based on mathematical modelling.

|  | **5 mg/kg** | | **6 mg/kg** | | **7·5 mg/kg** | | **10 mg/kg** | |
| --- | --- | --- | --- | --- | --- | --- | --- | --- |
|  | **Median** | **range** | **Median** | **range** | **Median** | **range** | **Median** | **range** |
| **150 ml/kg/day** | | | | | | | | |
| RID per day (average) (%) | 0·135 | 0·099-0·168 | 0·113 | 0·082-0·140 | 0·090 | 0·066-0·112 | 0·068 | 0·049-0·084 |
| RID_maximum day_ (%) | 0·749 | 0·473-0·936 | 0·624 | 0·394-0·780 | 0·499 | 0·315-0·624 | 0·374 | 0·236-0·406 |
| RID_d0-14_ (%) | 7·290 | 4·196-7·706 | 6·075 | 3·497-6·422 | 4·860 | 2·798-5·138 | 3·645 | 2·098-3·853 |
| RID_total exposure_ (%) | 10·138 | 7·414-12·599 | 8·449 | 6·178-10·499 | 6·759 | 4·943-8·399 | 5·069 | 3·707-6·299 |
| **200 ml/kg/day** | | | | | | | | |
| RID per day (average) (%) | 0·180 | 0·132-0·224 | 0·150 | 0·110-0·187 | 0·120 | 0·088-0·149 | 0·090 | 0·066-0·112 |
| RID_maximum day_ (%) | 0·998 | 0·630-1·248 | 0·832 | 0·525-1·040 | 0·665 | 0·420-0·832 | 0·499 | 0·315-0·624 |
| RID_d0-14_ (%) | 9·720 | 5·595-10·275 | 8·100 | 4·662-8·563 | 6·480 | 3·730-6·850 | 4·860 | 2·798-5·138 |
| RID_total exposure_ (%) | 13·518 | 9·886-16·798 | 11·265 | 8·238-13·999 | 9·012 | 6·590-11·199 | 6·759 | 4·943-8·399 |

#

**Supplementary file 5.**

**Table S2 Comparison of breast milk drug concentrations in high vs low volume milk, and in hindmilk vs foremilk.** Milk/Plasma (M/P) ratios are given for mixed milk disaggregated by total volume expressed at that sampling time point. Median and interquartile range are given for the time point at which the different volumes of milk were obtained to demonstrate differences (i.e. high milk volumes were expressed more in slightly earlier time points).

| **Milk volume** | | **Expected fat concentration** | **Number of sample time points** | **Time point, hours, median (IQR)** | **M/P ratio (IQR)** | **Ratio of HM and FM conc, median (IQR)** |
| --- | --- | --- | --- | --- | --- | --- |
| All | | - | 28 | 36 (20-168) | 0·89 (0·64-1·22)* | n.a. |
| <25 ml | | High | 11 | 36 (12-168) | 1·27 (1·02-1·51)* | n.a. |
| 25-75 ml | | Medium | 13 | 36 (20-336) | 0·68 (0·54-0·90)* | 1·79 (1·58-2·54) |
| >75 ml | | Low | 4^†^ | 28 (20-102) | 0·61 (0·55-0·70)* | 3·85 (3·02-5·53) |
| ≥25 ml | FM | Low | 17 | 36 (20-168) | 0·36 (0·27-0·65) | 2·42 (1·62-3·03) |
|  | MM | Medium | 17 | 36 (20-168) | 0·66 (0·54-0·89) |  |
|  | HM | High | 17 | 36 (20-168) | 0·96 (0·82-1·08) |  |

Abbreviations: FM foremilk, HM hindmilk, IQR interquartile range, ml millilitre, MM mixed milk

* using MM

^†^ these four samples came from two participants.

# References:

1. Anderson PO, Valdés V. Variation of Milk Intake over Time: Clinical and Pharmacokinetic Implications. Breastfeed Med. 2015 Apr;10(3):142–4.

2. Bowornkitiwong W, Komoltri C, Ngerncham S. The relationship between creamatocrit and cumulative percentage of total milk volume: a cross-sectional study in mothers of very preterm infants in Bangkok, Thailand. Int Breastfeed J. 2023 Nov 23;18(1):63.

3. Watson JA, Commons RJ, Tarning J, Simpson JA, Llanos Cuentas A, Lacerda MV, et al. The clinical pharmacology of tafenoquine in the radical cure of Plasmodium vivax malaria: An individual patient data meta-analysis. eLife. 2022 Dec 6;11:e83433.

4. Vélez ID, Hien TT, Green JA, Martin A, Sharma H, Rousell VM, et al. Tafenoquine exposure assessment, safety, and relapse prevention efficacy in children with Plasmodium vivax malaria: open-label, single-arm, non-comparative, multicentre, pharmacokinetic bridging, phase 2 trial. Lancet Child Adolesc Health. 2022 Feb;6(2):86–95.
